# Acute inflammatory response following lower-and upper-body Wingate anaerobic test in elite gymnasts in relation to iron status

**DOI:** 10.3389/fphys.2024.1383141

**Published:** 2024-07-10

**Authors:** Andrzej Kochanowicz, Tomasz Waldziński, Bartłomiej Niespodziński, Paulina Brzezińska, Magdalena Kochanowicz, Jedrzej Antosiewicz, Jan Mieszkowski

**Affiliations:** ^1^ Department of Gymnastics, Dance and Musical and Movement Exercises, Gdańsk University of Physical Education and Sport, Gdańsk, Poland; ^2^ Faculty of Health Sciences, University of Lomza, Łomża, Poland; ^3^ Faculty of Health Sciences and Physical Education, Kazimierz Wielki University, Bydgoszcz, Poland; ^4^ Department of Physical Therapy, Medical University of Gdansk, Gdańsk, Poland; ^5^ Department of Bioenergetics and Physiology of Exercise, Medical University of Gdansk, Gdańsk, Poland

**Keywords:** muscle damage, inflammation, oxidative stress, anaerobic exercises, male athletes, iron status

## Abstract

**Introduction:** Artistic gymnastics is one of the most demanding sports disciplines, with the athletes demonstrating extremely high levels of explosive power and strength. Currently, knowledge of the effect of gymnastic training adaptation on exercise-induced inflammatory response is limited. The study aimed to evaluate inflammatory response following lower- and upper-body high-intensity exercise in relation to the iron status in gymnasts and non-athletes.

**Methods:** Fourteen elite male artistic gymnasts (EAG, 20.6 ± 3.3 years old) and 14 physically active men (PAM, 19.9 ± 1.0 years old) participated in the study. Venous blood samples were taken before and 5 min and 60 min after two variants of Wingate anaerobic test (WAnT), upper-body and lower-body WAnT. Basal iron metabolism (serum iron and ferritin) and acute responses of selected inflammatory response markers [interleukin (IL) 6, IL-10, and tumour necrosis factor α] were analysed.

**Results:** EAG performed significantly better during upper-body WAnT than PAM regarding relative mean and peak power. The increase in IL-6 levels after upper-body WAnT was higher in EAG than in PAM; the opposite was observed after lower-body WAnT. IL-10 levels were higher in EAG than in PAM, and tumour necrosis factor α levels were higher in PAM than those in EAG only after lower-body WAnT. The changes in IL-10 correlated with baseline serum iron and ferritin in PAM.

**Discussion:** Overall, gymnastic training is associated with the attenuation of iron-dependent post-exercise anti-inflammatory cytokine secretion.

## Introduction

Artistic gymnastics is one of the most demanding sports disciplines. To stay competitive, the sports activity of elite professional gymnasts during the gymnastics season entails 5–6 h of gymnastic training per day, 6 days per week. The target training rigour starts at the early stages of the gymnast’s career (5–6 years old), with the training volume increasing yearly. In this scenario, gymnastic training exerts great physiological stress associated with neuromuscular and central fatigue, affecting body homeostasis and function. Multiple physiological and biochemical changes are induced in response to training, as evidenced by the associated inflammatory adaptation, metabolic changes, and recovery process kinetics (Alshammari et al., 2010). This type of adaptation is critical for realising such a demanding physical activity and is directly related to post-exercise stress tolerance (Kochanowicz et al., 2017).

In sports physiology, several biomarkers reflect the biochemical and physiological mechanisms underlying the physical stress induced by professional training (Marqués-Jiménez et al., 2016; Waldziński et al., 2023). For instance, determining myoglobin, creatine kinase, and lactate dehydrogenase serum levels is a golden standard for muscle damage analysis. Further, interleukin (IL) 1, IL-6, C-reactive protein, and tumour necrosis factor α (TNF-α) are considered specific inflammatory state markers (Pyne, 1994; Mieszkowski et al., 2021a; Waldziński et al., 2023). Their measurements are useful for obtaining a holistic overview of body function and muscle activity during competition and assessing an athlete’s body adaptation to specific exercise (Pyne, 1994) or training conditions (Ziemann et al., 2014). Every professional sports training event and sport activity is associated with increased inflammation and skeletal muscle tissue damage (Mieszkowski et al., 2021a). In addition to inflammation, oxidative stress markers, such as lipid and protein oxidation, are detected (Sohail et al., 2020). The excessive inflammatory response induced by exercise and driving the secretion of proinflammatory cytokines is considered one of the factors limiting sports performance (Fatouros et al., 2010).

Iron status profoundly influences inflammation, as systemic iron levels and homeostasis alterations affect the inflammatory response. Increased iron stores are associated with oxidative stress and inflammation (Kell, 2009). Interestingly, it has been observed that regular exercise reduces body iron stores and lowers oxidative stress and inflammation (Kortas et al., 2017).

One of the many factors that can influence exercise-induced inflammation may be serum iron and iron stored in human tissues. However, data on this subject are very limited; for example, iron status influences exercise-induced changes in adiponectin and myostatin (Kortas et al., 2020).

Acute exercise can induce a stress response in skeletal muscle and other tissue, which is manifested by the activation of stress-activated protein kinases (SAPK) (Parker et al., 2017). *In vitro*, experimental models demonstrated that activation of SAPK can lead to ferritin degradation, iron-dependent oxidative stress and proinflammatory response (Kell, 2009; Borkowska et al., 2011). Thus, it became reasonable to analyse whether iron status can influence inflammatory response after acute exercise tests.

The post-exercise iron homeostasis is regulated by several factors, but an essential role plays changes in the interleukin-6 (IL-6). This cytokine plays a regulator role in inflammation response and hepcidin secretion (Lee et al., 2005). Hepcidin is an amino acid peptide released by hepatocytes that is the predominant negative regulator of iron absorption in the small intestine and iron release from macrophages (Ganz, 2003). Moreover, its changes contribute to the regulation of inflammation, and many cytokines can stimulate hepcidin biosynthesis, leading to a decrease in serum iron. On the other hand, an increase in the labile iron pool within a cell can augment the activity of NfKB, which can lead to increased expression of proinflammatory cytokines (Kell, 2009).

In many sports disciplines, the physiological and biomechanical involvement of different body parts (e.g., the upper- and lower-body muscles) in physical activity is not the same (Bassa et al., 2002). Unfortunately, data on the associated differences are limited, as are those on the differences in the response to exercise of the upper and lower body (Kochanowicz et al., 2017). In the case of gymnasts, the overwhelming majority of exercise and training routines involve the upper body, e.g., activities involving supports, hanging, pushing, pulling up, or giving momentum to the rest of the body (Jemni et al., 2006; Sawicki et al., 2018; Kochanowicz et al., 2019). It was previously shown (Mieszkowski et al., 2021b) that in gymnasts, the upper-body anaerobic performance output is higher than that of the lower body compared to the untrained population and, thus, different biochemical adaptations in terms of the intensity of the inflammatory response and changes in iron metabolism elicited by the upper and lower body exercise could be expected. Demonstrating such relationships would verify whether the predominant gymnastic training methods, focused on using upper body parts, also affect systemic adaptive changes. Of note, any effort exclusively involving the upper parts of the body is often thought to induce only local adaptations because of the muscle body content.

The aim of the current study was to evaluate the changes in inflammatory response following lower- and upper-body high-intensity exercise in relation to the iron status in gymnasts and non-athletes.

## Materials and methods

### Experimental overview

In the current study, two study groups, athletes (elite gymnasts) and physically active controls, performed two variants of the maximal anaerobic effort: lower-limb and upper-limb exercises. Before and after exercise, the participants’ blood was collected, and inflammatory marker levels were analysed in the context of iron status.

### Participants

A group of 14 elite male artistic gymnasts (EAG, 20.12 ± 3.36 years old) and 14 physically active men (PAM, 20.18 ± 1.1 years old) participated in the study. The EAG group consisted of Polish professional gymnasts (training 6 times per week, 5–6 h per session) who compete on a senior level and are ranked in the International Gymnastics Federation classification. The PAM group consisted of volunteers (students) who declared regular participation in recreational sports, such as running, swimming, and team sports (on average, 2–3 times per week, 45–60 min per session).

All participants were considered healthy 6 months before the beginning of the study. Specifically, no bone or muscle tissue injuries were reported; with negative medical history regarding the cardiovascular, autonomic nervous system, or mental disorders, and any other condition that might have directly or indirectly affected the results. Further, no drugs or any other supplements were taken during the study.

Descriptive physical characteristics and basal (resting) levels of iron metabolism markers are presented in Table 1.

**TABLE 1:** Physical characteristics and basal values of iron metabolism markers.

Variable	Physically active men (n = 14)		Elite artistic gymnasts (n = 14)		*p*-value PAM/EAG
	Mean ± SD	(95% CI)	Mean ± SD	(95% CI)	
**Body height (cm)**	176.62 ± 4.87	174.56–180.10	170.44 ± 3.36*	168.10–172.25	<0.01
**Body mass (kg)**	72.24 ± 8.80	66.20–77.67	68.21 ± 5.80	64.78–72.12	0.16
**BMI (kg × m** ^**-2**^ **)**	23.34 ± 3.36	21.85–25.16	22.73 ± 1.76	21.54–24.05	0.54
**Percent body fat (%)**	10.88 ± 4.81	8.31–13.45	6.48 ± 2.97*	5.12–8.43	<0.01
**Iron (μmol/L)**	32.51 ± 7.85	27.97–37.05	26.51 ± 10.56	20.41–32.60	0.10
**Ferritin (ng/mL)**	134.72 ± 20.32	122.99–146.45	147.52 ± 33.96	127.92–167.13	0.23

Note: PAM, physically active men; EAG, elite artistic gymnasts; * significant difference between PAM, and EAG groups at *p* < 0.01.

### Experimental protocol

Before the experiment, the participants attended an orientation session to ensure they were familiar with the testing equipment and procedures. Their basic anthropometric characteristics were then measured. At least 3 months before the start of the study, all study participants refrain from taking any drugs and supplements that could influence obtained results (including preparations that could improve exercise capacity). Two days before the experiment, all participants were asked to refrain from extensive exercise, stay hydrated, and maintain their regular dietary habits, excluding any drugs and stimulants.

The experimental protocol comprised the measurement of maximal anaerobic effort using the Wingate anaerobic test (WAnT) to assess the adaptation of lower and upper limbs, with the load adjusted individually. All participants began with lower-body WAnT, and after a week’s break, they performed upper-limb WAnT. Before and after each WAnT session, blood samples were taken for further analysis. For each participant, the time of day, room temperature and other measurable variables were adequate during both WAnT performances (morning hours from 9 till 12 a.m., room temperature from 20 to 23°C, relative humidity: ≤70%, atmospheric pressure: 86 kPa–106 kPa).

### Lower-body and upper-body WAnT

The lower-body WAnT was conducted using a cycle ergometer (Monark 894E, Peak Bike, Sweden) according to the Bar-Or (Bar-Or, 1987). Each participant’s saddle height was adjusted individually (with the knee slightly flexed and with the final knee angle of approximately 170°–175°). Before any testing, each individual completed a standardised warm-up on the cycle ergometer (5 min at 60 rpm, 1 W/kg). During the testing, each participant was required to pedal for 30 s with a maximum effort against a fixed resistive load of 75 g/kg of total body mass.

The upper-body WAnT was conducted using a hand cycle ergometer (Monark 891E, Peak Bike) (Sawicki et al., 2018). Participants were seated in a chair, with the seat height and backrest adjusted individually. For the hand grasping the handles, the elbow joint was almost fully extended (140°–155°) (Kochanowicz et al., 2017). Similar to the lower-body WAnT, before any testing, the participants completed a warm-up that involved 5 min of arm cranking using a power output of 1 W/kg and a crank rate of 60 rpm. During the testing, each participant was required to pedal for 30 s with a maximum effort against a standard resistive load equivalent to 50 g/kg of total body mass. In both WAnTs, the procedure started without prior spinning of the flywheel due to the fact that in the specific nature of physical excesses, especially like gymnastics, the generation of maximum force values, regardless of the performed exercises, takes place always from a basic—static position. In a way, this seems to be much more reflected in the specificity of the physical effort than in the continuation of the effort that is already in progress.

During testing, verbal encouragement was given from the beginning until the end of the test to maintain the highest possible cadence throughout both WAnTs. Cycle ergometers were connected to a personal computer running the MCE 5.1 software (Staniak et al., 1994). The following WAnT variables were measured: peak power (W) and relative peak power (W/kg), calculated as the highest single point of power output (recorded at 0.2 s intervals), and mean power (W) and relative mean power (W/kg), calculated as the average power output during the 30 s test.

### Sample collection and measurements of inflammatory markers

Blood samples were collected at three time points by a medical diagnostic professional, according to the experimental protocol, i.e., before the test, immediately after (no more than 5 min after the test) and 60 min after the test. The blood was collected into 5 mL BD Vacutainer Clot Activator Tubes (Becton Dickinson and Company, NJ, United States). The serum was separated by centrifugation at 4,000 *g* for 10 min and aliquoted into 500 μL portions. The samples were frozen and stored (no longer than 6 months) at −80°C until further analysis.

Biochemical analysis of serum ferritin, IL-6, IL-10, and TNF-α levels were performed using high-sensitivity commercially available enzyme-linked immunosorbent assay kits (DRG International, Inc., Springfield, NJ, United States) and Thermo Fisher Scientific Elisa Analyzer (Thermo Fisher Scientific Waltham, MA, United States).

To assess baseline and changes in iron (FE) levels, plasma was collected into the lithium heparin tubes (Becton Dickinson and Company, NJ, United States) and tested using *in vitro* IRON 2 (Roche/Hitachi Cobas c.) systems using a Cobas C analyser 501.

Serum ferritin and iron levels were analysed only at baseline, as their concentrations are stable up to 24 h even after three repetitions of WAnT over a short period (Antosiewicz et al., 2013).

### Statistical analysis

Descriptive statistics included mean ± SD for all measured variables. The normality of distribution was checked using the Shapiro–Wilk’s test and Levene’s test was used to check the homogeneity of variance. As the assumptions of normality and homogeneity of variance were met, the analysis of variance (ANOVA) tests were used. One-way ANOVA was used to determine the difference in WAnT performance characteristics between the EAG and PAM groups.

To evaluate the changes in biochemical markers of inflammation and muscle damage before and after WAnT, two-way (2 × 3) ANOVA of repeated measures was performed, where group (GR) was the between-subject factor (EAG, PAM) and repeated measure (RM) was the within-subject factor (pre-WAnT, 5 min post WAnT, 60 min post WAnT). Pearson’s correlation coefficient was calculated between the baseline serum iron and ferritin levels and the changes (5 min vs. baseline; 60 min vs. baseline) in inflammatory marker levels.

The effect size of the participants’ characteristics (Cohen’s d-value) and biomarkers were determined using eta-squared statistics (ƞ^2^). In the analysis, ƞ^2^ values equal to or greater than 0.01 (d = 0.2; r = 0.1), 0.06 (d = 0.6; r = 0.3), and 0.14 (d = 0.8; r = 0.5) were the threshold values for a small, moderate, and large effect size, respectively (Cohen, 1988).

Power analysis for the interactions between the effects was performed using GPower ver. 3.1.9.2 to determine the appropriate sample size (Faul et al., 2007). Accordingly, for a medium effect size and test power of 0.80, the minimal required sample size was 28 participants.

All calculations and graphics were generated using GraphPad Prism 6.0 (GraphPad Software, MA, United States). All calculations were done using Statistica 12 (StatSoft, OK, United States). The level of significance was set at α = 0.05.

### Ethics

The study was approved by the Bioethics Committee for Clinical Research at the Regional Medical Chamber in Gdansk (decision no. KB-24/16) and carried out in accordance with the Declaration of Helsinki. All participants were informed about the purpose and test procedures, as well as the possibility of withdrawing consent at any time and for any reason. All participants gave written informed consent prior to the study.

## Results

In the current study, the body height and percent body fat of gymnasts were significantly lower than those of the controls (Table 1). However, body mass and resting iron and ferritin levels were not significantly different between the two groups (Table 1).

**FIGURE 1:**
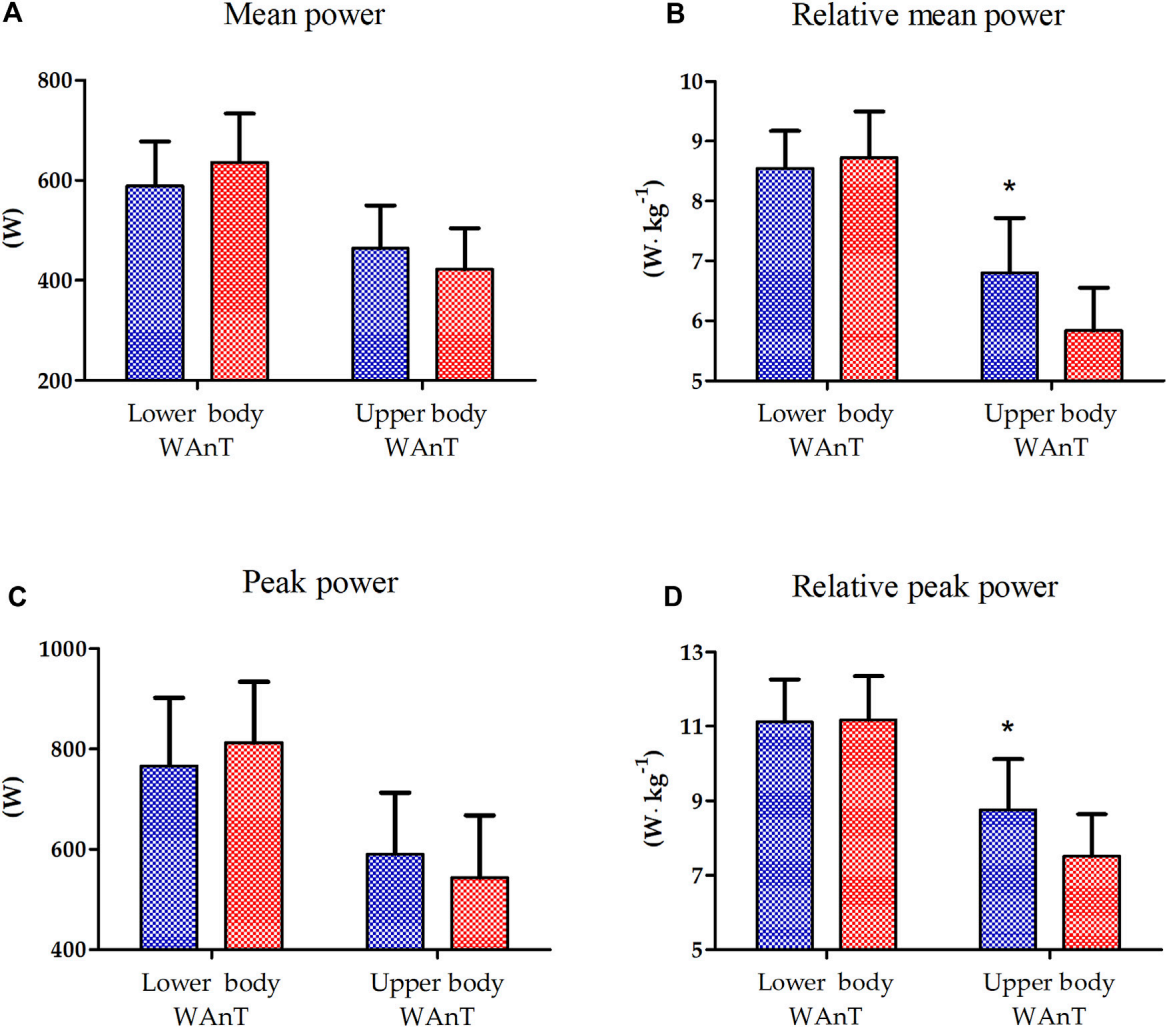
Lower and upper body Wingate Anaerobic Test (WAnT) characteristics in elite artistic gymnasts (blue, n = 14) and physically active men (red, n = 14). **(A)** mean power, **(B)** relative mean power, **(C)** peak power, **(D)** relative peak power. The data are presented as the mean and standard deviation; *p < 0.01, difference between elite artistic gymnasts and physically active men.

The results of one-way ANOVA of the absolute and relative peak power of lower- and upper-body WAnTs are presented in Figure 1. A significantly better performance of EAG was observed only for the relative mean (16.7%, *p* < 0.01) and peak power (15.5%, *p* < 0.05) generated during upper-body WAnT. Lower-body WAnT results for both groups were not statistically different (Figure 1).

**FIGURE 2:**
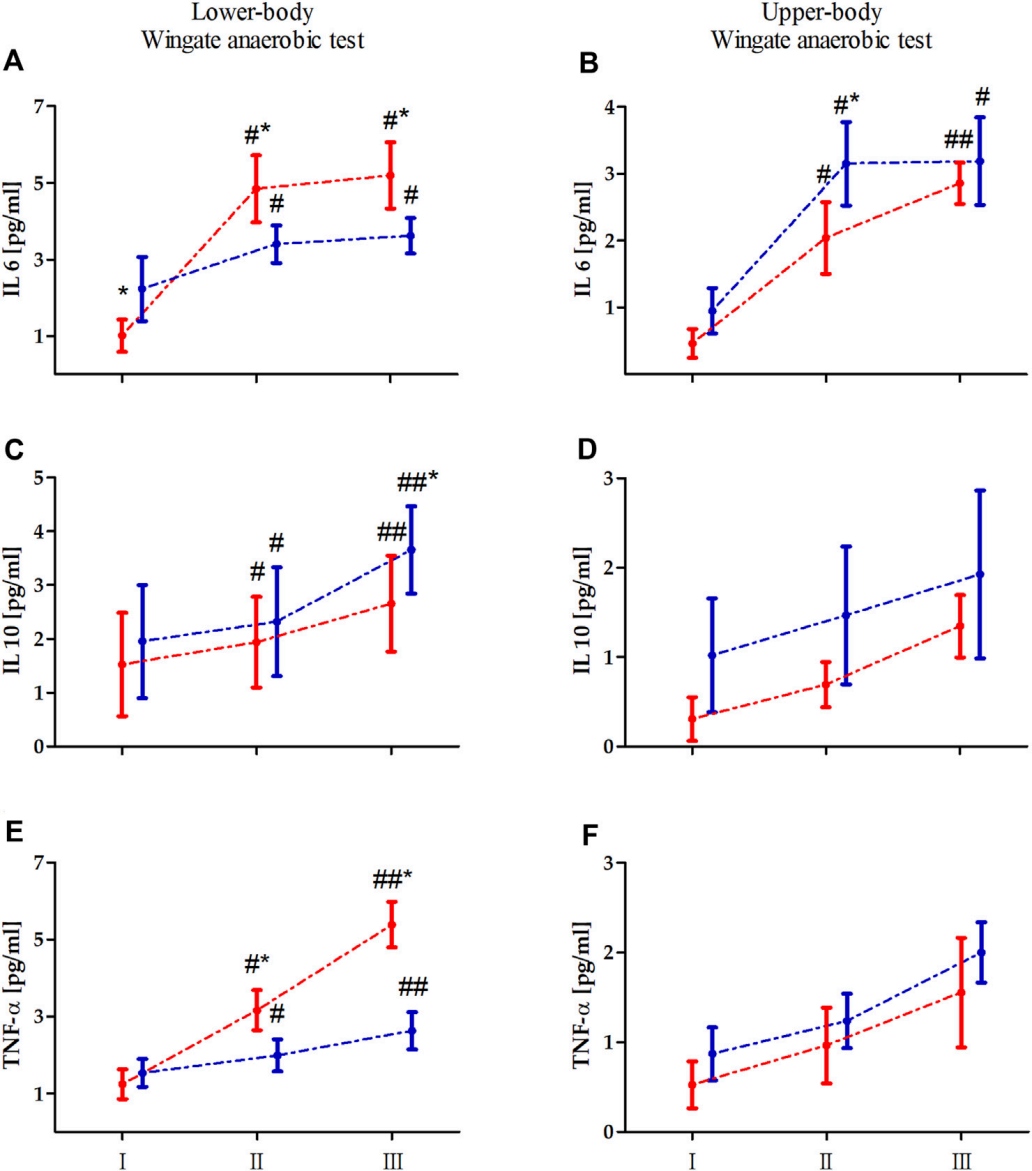
Changes in the levels of interleukin (IL) 6, IL-10, and tumor necrosis factor alpha (TNF-α) induced by lower **(A, C, E)** an upper-body **(B, D, F)** Wingate anaerobic test (WAnT) in elite artistic gymnasts (blue, n = 14) and physically active men (red, n = 14). Vertical bars, mean and standard deviation; I, resting value; II, 5 min after WAnT; III, 60 min after WAnT. Statistical analysis: * difference between elite artistic gymnasts and physically active men at particular time point at *p* < 0.01; # significant difference vs. rest value in particular group at *p* < 0.01, ## significant difference vs. rest value and 5 min after the WAnT in particular group at *p* < 0.01.

Two-way ANOVA with repeated measures of exercise-induced inflammation during lower- and upper-body anaerobic exercise is shown in Table 2. The analysis of lower- and upper-body WAnT data revealed a significant effect of RM on each tested inflammatory marker. The GR effect of the tested markers was also significant for both types of WAnT, except for the effect on changes in IL-10 levels after lower-body WAnT. Of note, IL-6 and TNF-α levels after lower-body WAnT in EAG were significantly lower than those in PAM; on the other hand, upper-body WAnT induced significantly higher levels of IL-6 and TNF-α in EAG than in PAM. Post-hoc analysis of changes in IL-10 levels induced by upper-body WAnT also revealed significantly higher readings in EAG. Considering the interaction of GR and RM factors, while the IL-6 and TNF-α levels in EAG were significantly lower than those in PAM 5 min (IL-6, 29.7%, *p* < 0.01; TNF- α, 37.3%, *p* < 0.01) and 60 min (IL-6, 29.7%, *p* < 0.01; TNF-α, 51.3%, *p* < 0.01) after lower-body WAnT, the IL-10 levels 60 min after exercise were significantly higher in EAG than those in PAM (27.7%, *p* < 0.05) (Figure 2). On the other hand, the IL-6 levels 5 min after upper-body WAnT were significantly higher in EAG than those in PAM (55.17%, *p* < 0.01).

**TABLE 2:** Two-way (two groups × three repeated measurements) ANOVA of the secretion of specific cytokines induced by lower- and upper-body anaerobic exercise in elite artistic gymnasts and physically active men.

*Variable*	*Exercise*	*Effect*	*F*	*Df*	*p*	*Effect size (η^2^)*	*Post-hoc outcome*
*IL-6*	Lower	GR	6.83	1, 26	0.01*	0.20	EAG < PAM
	body	RM	402.10	2, 52	0.01**	0.93	III > II > I
	WAnT	GR × RM	106.05	2, 52	0.01**	0.80	II^EAG^ < II^PAM^;
							III^EAG^ < III^PAM^
	Upper	GR	16.25	1, 26	0.67	0.01	EAG > PAM
	body	RM	300.97	2, 52	0.01**	0.91	III > II > I
	WAnT	GR × RM	10.25	2, 52	0.01**	0.27	II^EAG^ > II^PAM^
*IL-10*	Lower	GR	3.67	1, 26	0.07	0.11	
	body	RM	150.78	2, 52	0.01**	0.85	III > II > I
	WAnT	GR × RM	8.35	2, 52	0.01**	0.24	III^EAG^ > III^PAM^
	Upper	GR	0.83	1, 26	0.36	0.03	EAG > PAM
	body	RM	75.28	2, 52	0.01**	0.73	III > II > I
	WAnT	GR × RM	2.45	2, 52	0.09	0.08	
*TNF-α*	Lower	GR	61.96	1, 26	0.01*	0.70	EAG < PAM
	body	RM	613.68	2, 52	0.01**	0.95	III > II > I
	WAnT	GR × RM	207.48	2, 52	0.01**	0.88	II^EAG^ < II^PAM^;
							III^EAG^ < III^PAM^
	Upper	GR	8.52	1, 26	0.01**	0.24	EAG > PAM
	body	RM	112.75	2, 52	0.01**	0.81	III > II > I
	WAnT	GR × RM	0.72	2, 52	0.48	0.02	

Note: IL-6, interleukin 6; IL-10, interleukin 10; TNF-α, tumour necrosis factor α; Df, degrees of freedom, where a first and second number are variability between and withing groups, respectively; GR, group; RM, repeated measure; PAM, physically active men (n = 14); EAG, elite artistic gymnasts (n = 14); I, resting value; II, 5 min after 30 s upper- or lower-body, as indicated, Wingate anerobic test (WAnT); III, 60 min after 30 s upper- or lower-body, as indicated, WAnT; * significant differences at *p* < 0.05, ** significant differences at *p* < 0.01.

The results of the correlation analysis of changes in the IL-6, IL-10, and TNF-α levels induced by the upper- and lower-body anaerobic exercise with the baseline (resting) serum levels of iron and ferritin are presented in Tables 3, 4, accordingly. The resting iron levels showed a significant positive correlation with changes in the IL-10 levels in PAM (Table 3). Similarly, the correlation analysis of baseline ferritin levels revealed a significant positive correlation with the change in IL-10 levels 60 min after lower-body WAnT in PAM only.

**TABLE 3:** Correlation of changes in specific cytokine levels induced by upper- and lower-body Wingate anaerobic test with baseline iron serum levels.

*Variable*	*Time point* *(min)*	*Lower-body Wingate anaerobic test*			*Upper-body Wingate anaerobic test*		
		PAM	EAG	All	PAM	EAG	All
*IL-6*	Delta 5	−0.39	0.01	0.22	0.01	−0.02	−0.19
	Delta 60	−0.50	0.14	0.24	0.09	0.15	0.19
*IL-10*	Delta 5	0.83*	−0.12	0.06	0.45	0.29	0.27
	Delta 60	0.78*	−0.19	−0.06	0.29	0.17	0.23
*TNF-α*	Delta 5	0.03	0.14	0.32	0.36	−0.20	0.07
	Delta 60	−0.13	0.51	0.35	0.31	−0.20	0.02

Note: PAM, physically active men (n = 14); EAG, elite artistic gymnasts (n = 14); IL-6, interleukin 6; IL-10, interleukin 10; TNF-α, tumor necrosis factor α; * significant correlation at *p* < 0.05.

**TABLE 4:** Correlation of changes in specific cytokine levels induced by upper- and lower-body Wingate anaerobic test with baseline ferritin serum levels.

*Variable*	*Time point* *(min)*	*Lower-body Wingate anaerobic test*			*Upper-body Wingate anaerobic test*		
		PAM	EAG	All	PAM	EAG	All
*IL-6*	Delta 5	−0.22	−0.22	−0.30	0.24	0.29	0.35
	Delta 60	−0.17	−0.32	−0.32	−0.08	0.20	0.09
*IL-10*	Delta 5	0.50	−0.04	0.03	0.19	−0.34	−0.16
	Delta 60	0.76*	0.11	0.36	0.12	−0.24	−0.18
*TNF-α*	Delta 5	0.47	0.12	−0.14	0.18	−0.04	0.01
	Delta 60	−0.14	−0.16	−0.26	0.30	−0.05	0.12

Note: PAM, physically active men (n = 14); EAG, elite artistic gymnasts (n = 14); IL-6, interleukin 6; IL-10, interleukin 10; TNF-α, tumor necrosis factor α; * significant correlation at *p* < 0.05.

## Discussion

The aim of the current study was to evaluate and compare the inflammatory response (i.e., changes in IL-6, IL-10, and TNF-α levels) after lower- and upper-body high-intensity exercise in gymnasts and non-athletes in relation to iron status.

To the best of our knowledge, this is the first study in which the effect of upper- and lower-body anaerobic exercise on the inflammatory state markers was compared in professional athletes and non-athletes in the context of iron status. The main outcome of the study is that the lower- and upper-body maximal exercise in the form of WAnT elicited different responses of inflammatory markers depending on the training status. Specifically, the increase in IL-6 levels was more pronounced in EAG after upper-body WAnT, while it was more pronounced in PAM after lower-body WAnT. The other observed differences, i.e., relatively higher IL-10 levels in EAG and relatively higher TNF-α levels in PAM, were only associated with lower-body WAnT.

The study revealed that the relative peak (15.46%) and mean (16.72%) power of the upper body during WAnT are significantly higher in EAG than those in PAM. That is mainly because of the specific adaptation induced by and observed in professional gymnastic training. Namely, gymnasts mainly engage the upper body muscles to perform most of their exercises during the competition (e.g., floor exercises, parallel bars, horizontal bars, and others), with the lower body mainly engaged in short, explosive efforts (e.g., jumping). Such discrepancy in the upper- and lower-body WAnT performance in gymnasts has been reported before (Jemni et al., 2006). This may help to explain the lack of differences in the relative peak and mean power between EAG and PAM observed for lower-body WAnT and could be a starting point for understanding the differences in the response of inflammatory markers in the two groups.

Exercise initiates a cascade of inflammatory events, which affect human health in the long term. During and after acute exercise of the skeletal muscle, interactions between immune cells, cytokines, and other intracellular components create an inflammatory milieu responsible for the recovery from an adaption to an exercise bout. One of the main cytokines responsible for regulating the inflammatory process is IL-6, essential in initiating and controlling the post-exercise inflammatory process. Typically, physical exercise is accompanied by increased IL-6 levels due to the onset of inflammation (Aaseth and Birketvedt, 2012; Antosiewicz et al., 2013).

In the current study, analysis of the tested inflammatory markers revealed that considering the nature of the maximal anaerobic exercise, the IL-6 and TNF-α levels after lower-body WAnT in gymnasts were significantly lower than those in the controls. In comparison, the IL-6 levels induced by upper-body anaerobic exercise were significantly higher in gymnasts than in the controls. Typical physical training-induced adaptation leads to a decrease in basal and acute post-exercise IL-6 production is observed. This is related to the counteractive effects of several potential stimuli of IL-6 (Keller et al., 2001; Pedersen et al., 2001; Fischer, 2006). In the current study, we observed that the resting (before worm up) IL-6 levels, especially in the case of lower-body WAnT in EAG, were increased. This may be associated with two factors. First, the gymnasts are professionals, training 6 times per week, 5–6 h per session. Second, the emotional reaction to the test performance should be considered. As mentioned, gymnasts are very well prepared for upper body testing because of the specificity of their training. However, lower-body WAnT is not the type of physical effort characteristic for their sports training. It may be associated with excessive adrenergic activation, which also increases IL-6 levels (Rodas et al., 2020). The observed differences were not statistically significant in meters of maximal and mean power achievements. Furthermore, the analysis of IL-10 levels induced by upper-body anaerobic exercise revealed significantly higher readings among gymnasts. IL-10 is an anti-inflammatory cytokine whose blood levels mainly increase after exercise (Stankiewicz et al., 2023). Accordingly, while we observed a significant increase in the IL-10 levels after upper- and lower-body WAnT in EAG and PAM, the increase was more pronounced for the former. This may suggest that the anti-inflammatory response in gymnasts is more pronounced than that in non-athletes because of their long-term training (Marciniak et al., 2009; Kajaia et al., 2018). Finally, it has been reported that the IL-6 level increase in response to exercise prevents a subsequent increase in the levels of pro-inflammatory cytokines, such as TNF-α (Petersen and Pedersen, 2005) and induces the production of IL-10 (Steensberg et al., 2003), conferring anti-inflammatory properties to that response (Pedersen et al., 2003). This aligns with the observations in the current study.

Inflammation is profoundly influenced by iron status, as alterations of systemic iron levels and homeostasis affect the inflammatory response. Increased iron stores are correlated with increased secretion of inflammatory response markers (Kell, 2009). However, we are unaware of any study that has studied the role of iron in acute exercise-induced inflammation.

The current study analysed iron status, interleukin (IL-6 and IL-10), and TNF-α secretion revealed differences in PAM and EAG upon lower- and upper-body exercise. Each type of intensive exercise induces physiological stress that can contribute to an increased formation of free radicals. Body iron stores can modulate this process. One of the training adaptations associated with systemic radical formation and iron metabolism is the reduction of ferritin levels (body iron stores) (Lakka et al., 1994; Kortas et al., 2017). This type of adaptation may be related to lower oxidative stress caused by decreased iron-dependent free radical formation (Nemeth et al., 2004; Kortas et al., 2017). Furthermore, it has been shown that oxidative stress (which can be iron-dependent) induced during intensive exercise leads to increased levels of pro-inflammatory cytokines, such as IL-6 and TNF-α (Suzuki, 2018). This may explain why a reduction in body iron stores, which may be observed as a training adaptation, would contribute to reducing oxidative stress and inflammation caused by physical activity (Kortas et al., 2017).

In the present study, the basal serum-iron levels in EAG were lower than in PAM (26.51 ± 10.56 vs. 32.51 ± 7.85 (μmol/L)). While the difference was not statistically significant, it may be associated with long-term gymnastic training. Further, we observed that in PAM, the baseline serum levels of iron and ferritin were highly correlated with the changes in IL-10 levels induced by lower-body WAnT. In addition to higher IL-6 and TNF-α levels, it indicates a higher post-exercise inflammation in non-athletes and a possible modulatory role of iron in this process (Stankiewicz et al., 2023). Serum iron can have signalling properties as it can enter cells through the transferrin receptor, which can lead to an increase in the labile iron pool (LIP). An increase in LIP can lead to the activation of NFKB, a transcriptional factor that can augment the transcription of genes encoding proinflammatory cytokines like IL-6 and TNF. Conversely, IL-6 has been proposed to induce the expression of antiinflammatory cytokines, including Il-10. Here, we observed that lower-body exercise test-induced changes in IL-10 are strongly correlated with serum iron, confirming its signalling role. Similarly, we observed that ferritin concentration correlates with exercise-induced changes in IL-10. Ferritin iron is considered inert as it does not stimulate free radicals’ formation. However, studies on cell culture demonstrated that during stress conditions, part of ferritin undergoes ferritin degradation, which can lead to an increase in LIP (Borkowska et al., 2011). The degradation process depends on c-jun terminal kinase (JNK), which belongs to stress-activated protein kinases (Antosiewicz et al., 2007). If we consider that JNK activation can be blunted by heat shock proteins (HSP), their higher levels can influence the reaction to exercise. Regular exercise has been shown to upregulate HSP (Febbraio and Koukoulas, 2000). Thus, we can speculate that our athletes are much more resistant to exercise-induced activation of JNK. Fewer possibilities can increase LIP in skeletal muscle and other tissue. This can be a likely explanation for why, in EAG, there is no correlation between ferritin and changes in serum cytokines.

The current study has some limitations. Specifically, although we evaluated the acute inflammatory response induced by upper- and lower-body WAnT, we focused on only a few well-known parameters contributing to the inflammatory status. This approach may not fully reflect the complexity of the adaptation process induced by many years of training, especially considering the molecular and physiological aspects of the process. On the other hand, we have previously reported that gymnastic training may induce some adaptations on the molecular level, impacting the expression of inflammatory genes (those encoding IL-6 and IL-10) and genes encoding heat-shock proteins HSPA1A and HSPB1 (Kochanowicz et al., 2017; Zychowska et al., 2017). In the current study, we confirmed the observed molecular adaptations, manifesting as changing serum levels of the related inflammatory markers.

## Conclusion

We here showed that gymnastic training significantly affects the post-exercise inflammatory response and that the response is lower- and upper-body WAnT-dependent. This effect is most likely a result of many years of specific training focused on various upper-body muscle groups and the explosive muscle strength of the lower body, which induces physiological and biochemical adaptations to exercise. Further, the presented findings suggest that exercise-induced pro- and anti-inflammatory cytokine production, essential for body homeostasis, may depend on body iron storage and serum iron.

Analysis and evaluation of post-exercise secretion of pro- and anti-inflammatory cytokines in relation to iron status may be a useful indicator of exercise adaptation, and showing the health benefits of sports training and complexity of the body’s response to exercises.

## Data Availability

The raw data supporting the conclusions of this article will be made available by the authors, without undue reservation.
